# Association between severe unaddressed dental needs and developmental health at school entry in Canada: a cross-sectional study

**DOI:** 10.1186/s12887-019-1868-x

**Published:** 2019-12-07

**Authors:** Magdalena Janus, Caroline Reid-Westoby, Catherine Lee, Marni Brownell, Jonathon L. Maguire

**Affiliations:** 1 Offord Centre for Child Studies, Department of Psychiatry and Behavioural Neurosciences, McMaster University, McMaster Innovation Park, 175 Longwood Rd South, Suite 201A, 1280 Main St. West, Hamilton, ON L8S 4K1 Canada; 2 Bachelor of Health Sciences (Honours), Faculty of Health Sciences, McMaster University, Hamilton, ON Canada; 3 Manitoba Centre for Health Policy, Department of Community Health Sciences, University of Manitoba, Winnipeg, MB Canada; 4 Department of Pediatrics, Faculty of Medicine, University of Toronto, Toronto, ON Canada; 5 Department of Nutritional Sciences, Faculty of Medicine, Universtiy of Toronto, Toronto, ON Canada; 6 Li Ka Shing Knowledge Institute, St. Michael’s Hospital, Toronto, ON Canada; 7 Department of Paediatrics, St. Michael’s Hospital, Toronto, ON Canada

**Keywords:** Early Development Instrument, Teacher-reported unaddressed dental needs, Developmental health at school entry

## Abstract

**Background:**

Dental problems are the most prevalent chronic disease worldwide, with up to half of all kindergarten children having tooth decay. However, there is a lack of evidence of whether unaddressed dental needs (UDNs) are associated with children’s developmental health, a concept reflecting holistic child development - encompassing physical, emotional, and cognitive development. The objective of the current study was to evaluate the relationship between UDNs and developmental health among kindergarten children using the Early Development Instrument (EDI).

**Methods:**

We examined associations between teacher reported UDNs and developmental vulnerability on the EDI. Children were included in the study if they were enrolled in kindergarten in publicly-funded schools in Canada between 2010 and 2015, had been in the classroom for at least one month, and had no more than 25% of missing items on the questionnaire.

**Results:**

Among 576,264 children who met inclusion criteria (95.4% of eligible children), 2465 (0.4%) were identified as having UDNs by their teachers. Children with UDNs had 4.58 to 8.27 times higher odds of being vulnerable on any of the five developmental domains (physical health and well-being, social competence, emotional maturity, language and cognitive development, communication skills and general knowledge), compared to children without UDNs.

**Conclusion:**

In this study, teacher-reported UDNs were associated with developmental vulnerability in kindergarten children. Teacher reported unmet dental needs in kindergarten children may be a proxy for poor developmental health at school entry, and thus a marker for supporting both children’s oral health and early developmental needs.

## Background

Poor oral health is a worldwide public health problem, with millions of children experiencing caries in their primary teeth [1]. Estimates suggest that 60 to 90% of school-aged children have some form of tooth decay [2] and in Canada, up to half of all children enter kindergarten with tooth decay [3, 4]. While tooth decay is common across the globe [5], it is also a marker for health inequalities, with people of lower socioeconomic status (SES) experiencing poorer oral health [6–9]. Poverty is related to a higher risk of dental caries, unaddressed dental needs (UDNs), and poor oral health-related quality of life [10]. UDNs are oral health issues, such as dental caries, that have not yet been treated or corrected. In the United States, over 40% of low-income individuals 20 to 64 years of age had untreated dental caries between 2005 and 2008, compared to 16% of high-income people [11]. A Canadian study found a strong SES-based inequity in preventive dental care utilization, with lower-income individuals tending to postpone visits to the dentist [12, 13]. In addition to the inequalities in oral health between the lower and higher SES groups, there is also evidence of a social gradient in oral health [14, 15], where the difference in outcomes is gradual and exists along the full spectrum of SES. UDNs have also been associated with special health needs (SHNs) in children. Children with SHNs are defined as having either a disability, exceptionality, or a functional impairment, such as a visual or hearing impairment, and they typically require additional assistance in the classroom [16]. Research indicates that they have poorer oral hygiene and a greater incidence of caries (both treated and untreated), as well as other oral diseases compared to their non-SHNs peers [17, 18], with reports of 20% of children with a SHN having UDNs [19]. It has been suggested that a lack of training for dental professionals on how to treat children with SHNs [20] is one of the reasons for their poorer oral health, as some of them, such as children with Autism Spectrum Disorder (ASD), appear to face multiple barriers in accessing dental care [21].

Dental caries in young children have been associated with various aspects of their health. For instance, children with caries report experiencing pain [22, 23], impacting their ability to eat and sleep, participate in school activities and learn [23–25]. Dental caries also affect children’s nutrition and growth which are associated with poorer developmental outcomes. Acs and colleagues [26] found that 3-year-old children with severe dental caries weighed on average 1 kg less than children without caries. Other studies have found an association between dental health and academic achievement [27–29], as well as psychosocial well-being [22, 25, 28]. Little is known about the association between oral health and children’s developmental health. Developmental health is a concept put forth by Keating and Hertzman [30] that is based on the framework of social determinants of health [31], and is meant to encompass biological aspects of health (i.e., physical), as well as behavioural (emotions, cognitive skills). In doing so, it promotes a holistic view of early childhood, beyond simplistic cognitive-only school readiness or absence of illness [32]. The Early Development Instrument (EDI) is a population-level measure of children’s developmental health at school entry which has been used globally and been well validated [33–36].

While dental caries and tooth decay are common problems among children, little is known about how teacher identification of UDNs may be related to children’s developmental health at school entry. For children with limited access to dental health care in particular, teacher’s observation of dental needs may be an important marker of overall health needs. We hypothesized that children with UDNs would have a greater chance of having developmental vulnerability than children without such needs. The primary objective of this observational, cross-sectional study was to evaluate the association between teacher-reported UDNs in Canadian kindergarten children and developmental health at school entry based on the total EDI score. Secondary objectives were to examine the association between teacher-reported UDNs and any area of developmental vulnerability on the EDI including physical, socioemotional, language/cognitive, and communication and general knowledge.

## Methods

### Study design

A cross-sectional, population-level study of kindergarten children with teacher-identified UDNs, as reported on the EDI, was carried out in Canada between 2010 and 2015.

### Study population

The study population consisted of 603,904 kindergarten children attending publicly-funded schools between the 2009/2010 and 2014/2015 school years from most Canadian provinces and territories (see Table 1). Based on population estimates [37], approximately 90% of children living in Canada attend publicly-funded kindergarten [38]. These children were part of a population-level study of developmental outcomes of kindergarten children with health disorders, referred to as the *C*anadian *C*hildren’s *H*ealth *i*n *C*ontext *S*tudy (CCHICS) [39]. The aim of the CCHICS was to establish a pan-Canadian database for monitoring developmental health and well-being of children with health disorders. The CCHICS merged pan-Canadian EDI data with neighbourhood-level SES data (see below for description of the measures). All children who met the following criteria were included in the study: 1) were enrolled in kindergarten; 2) were in their current classroom for at least one month; and 3) had a questionnaire with no more than 25% of items missing.

**Table 1:** Implementation of the EDI in Canada between 2009/2010 and 2014/2015

Province	2009/2010	2010/2011	2011/2012	2012/2013	2013/2014	2014/2015
Alberta	21,976	20,881	14,492	20,734	–	–
British Columbia	25,033	21,911	12,485	30,034	1289	–
Manitoba	–	12,437	–	13,538	–	13,776
New Brunswick	–	–	–	–	–	–
Newfoundland & Labrador		1106	2135	4942	5182	
Northwest Territories	–	–	672	659	654	645
Nova Scotia	913	2402	2257	8592	1397	8677
Nunavut	–	–	–	–	–	–
Ontario	33,305	38,728	57,038			135,936
Prince Edward Island	–	–	–	–	–	–
Quebec	–	–	65,498	–	–	–
Saskatchewan	8625	5501	552	8427		
Yukon	362	344	368	401	–	–

### Measures

The primary exposure variable was teacher-reported UDNs. This was measured by teachers’ answers to the following question: “Does the student have a problem that influences his/her ability to do schoolwork in a regular classroom? If yes, please mark all that apply.” There were 11 different options, one of which was “unaddressed dental needs” (see Table 2). This option was added to the questionnaire in 2010, as a response to a review of comments from teachers received in the previous 6 years of implementation of the EDI, which indicated that dental needs were considered as an impairment of children’s ability to participate. A small group of kindergarten teachers provided feedback on the usefulness and wording of the item, indicating that it was a feasible and useful addition. It should be noted that the UDNs reported by teachers most likely represent the most severe cases as it is improbable that minor or even moderate dental caries would affect children’s ability to function in a regular classroom. As such, the prevalence of UDNs reported in our study is presumably an underestimate of the true prevalence of UDNs among 5-year-olds.

**Table 2:** Functional impairments available on the EDI

Does this child have a problem that influences his/her ability to do schoolwork in a regular classroom?	
a. Physical disability	
b. Visual impairment	
c. Hearing impairment	
d. Speech impairment	
e. Learning disability	
f. Emotional problem	
g. Behavioural problem	
h. Home environment/problems at home	
i. Chronic medical/health problems	
j. Unaddressed dental needs	
k. Other	

The primary outcome was vulnerability in any of the developmental domains of the EDI (i.e., vulnerable on one or more domains). Secondary outcomes were vulnerability in each of the five developmental domains of the EDI: physical health and well-being, social competence, emotional maturity, language and cognitive development, and communication skills and general knowledge. Covariates included variables which are known or suspected to be potential confounders of the relationship between oral health and child development, identified through an extensive literature review. These included child’s age, sex, special needs designation, having English or French as a second language (E/FSL), and area-level SES.

#### Early Development Instrument

The EDI is a 103-item, teacher-completed questionnaire that measures children’s ability to meet age-appropriate developmental expectations prior to entering Grade 1 [33]. The EDI is completed in the second half of the school year by kindergarten teachers for each student in their class. The EDI measures five general domains of development: physical health and well-being, social competence, emotional maturity, language and cognitive development, and communication skills and general knowledge. In addition to measuring children’s development, the EDI collects demographic information for each child, such as their date of birth, sex, whether they have E/FSL (yes or no), and whether they have been identified as having a special need (yes or no; this is a school-based designation that identifies children with a chronic condition or those who require additional assistance in the classroom) [33]. Since 2010, the EDI has collected information on whether children have UDNs, either through information provided by the parents or through teacher observations. Teachers can select either of these options or both; the response options are combined into one dichotomous variable (UDNs, yes/no).

There are two main EDI outcomes: overall vulnerability and individual domain scores. Domain scores are an average of the items that contribute to each domain, which vary from 0 to 10, with a higher score indicating greater ability [33]. The EDI scores for each domain are divided into categories representing the highest to the lowest scores in the given population. The distribution of scores across each of the five domains are utilized to determine percentages of children at various levels of developmental health. Children scoring below the 10th percentile in one or more of the five domains are categorized as “vulnerable” in terms of their developmental health based on national standards [40]. The EDI has been used extensively throughout Canada, Australia, and other parts of the world [41]. Over the past decade, several studies have examined different psychometric properties of the EDI, including between-group reliability [34], construct validity [35], cross-cultural validity [36], and predictive validity [42]. The results have consistently shown that the EDI is valid and reliable and can be reliably used as a measure of early child developmental health. The internal consistencies, using Cronbach’s alphas for each domain were .78 for physical health and well-being, .96 for social competence, .93 for emotional maturity, .91 for language and cognitive development, and .94 for communication skills and general knowledge.

#### Neighbourhood-level SES

Information on neighbourhood-level SES was retrieved from the 2010 Taxfiler databases and the 2011 National Household Survey which are national Canadian surveys collected through Statistics Canada [42]. An SES index identifying 10 developmentally-relevant socioeconomic variables was created for 2058 custom-defined neighbourhoods across the country. The index measures aspects related to household income, education, mobility, immigration, single parenthood, and first language [43]. The SES index was transformed into Z-scores, with a mean of 0 and a standard deviation of 1. The neighbourhood SES index was merged with the EDI dataset using children’s postal codes with a 99.3% match rate.

### Analytical strategy

UDNs were examined, comparing the percentages of children with UDNs, as reported by their teacher, in each of the provinces/territories included in the study. Descriptive statistics including means and proportions were examined for children with and without UDNs. Children’s age, sex, special needs designation, E/FSL, and area-level SES were compared between children with and without UDNs using contingency tables. For the primary analysis, a binary logistic regression (BLR) model was developed to determine the association between UDNs and overall vulnerability in any of the developmental domains (i.e., vulnerable on one or more domains), while controlling for the pre-specified potential confounding variables mentioned above. For the secondary analysis, if the association between UDNs and overall vulnerability was statistically significant (*p* < 0.05) then an additional five BLR models were constructed to examine the association between each specific EDI domain, as mentioned above, with adjustment for the same potential confounding variables as the primary model. To account for multiple hypothesis testing, the level of statistical significance for each secondary analysis was set at *p* < 0.01. All statistical analyses were conducted using the statistical software SPSS, version 25 [44]. CCHICS has been approved by the Hamilton Integrated Research Ethics Board and the University of Manitoba Health Research Ethics Board.

## Results

### Sample characteristics

**Fig. 1:**
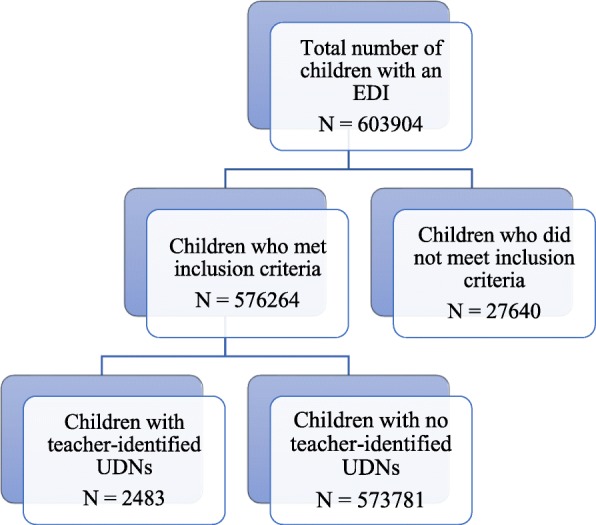
Flowchart of participants

**Fig. 2:**
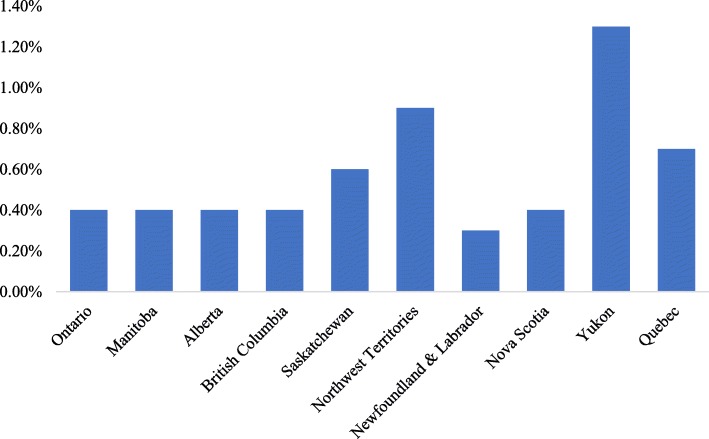
Percentage of children with UDNs, by province

A total of 576,264 children (95.4% of the eligible study population) met the inclusion criteria and were included in the analyses. A total of 2483 children (0.4% of the final analytical sample) were identified as having UDNs (see Fig. 1). Figure 2 displays the number and percentages of children with teacher-reported UDNs by province. Table 3 presents demographic characteristics of children with and without UDNs. Children with teacher-reported UDNs were similar in age but more likely to be male, have a special needs designation, have E/FSL, and have lower neighbourhood-level SES than children without teacher-reported UDNs. These differences were all statistically significant at *p* < .001.

**Table 3:** Description of included children with and without teacher identified UDNs

Variables	Children with UDNs	Children without UDNs
	Mean (SD)	Mean (SD)
Age at EDI completion (years)	5.77 (0.39)	5.71 (0.32)
Neighbourhood-level SES (z-score)	−0.26 (1.01)	0.12 (1.03)
	Number (%)	Number (%)
Males	1495 (60.4%)	293,743 (51.2%)
Children with special needs	460 (18.6%)	20,301 (3.5%)
Children with E/FSL	489 (19.7%)	74,872 (13.1%)

### Association between UDNs and children’s developmental health

**Fig. 3:**
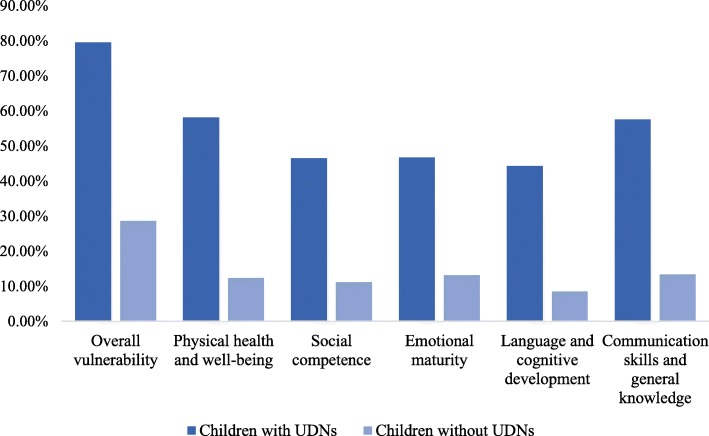
Percentage of children vulnerable overall and on each EDI domain, by presence of UDNs

Figure 3 presents vulnerability rates on one or more domains, as well as each of the five domains, for children with and without UDNs. In the primary analysis, teacher-reported UDNs were associated with overall vulnerability on the EDI in both unadjusted (*p* < 0.001) and adjusted models controlling for age, sex, special needs, E/FSL, and neighbourhood-level SES (*p* < 0.001). Children with teacher-reported UDNs had 8.4 times higher odds of being vulnerable on at least one domain of the EDI compared to children without UDNs (see Table 3). In the secondary analyses, in both unadjusted and adjusted models, teacher-reported UDNs were associated with vulnerability on each EDI developmental domain (*p* < 0.001). Controlling for the potential confounding variables mentioned above, children with UDNs had 8.3 times higher odds of being vulnerable in physical health and well-being, 5.4 times higher odds of being vulnerable in social competence, 4.6 times higher odds of being vulnerable in emotional maturity, 6.6 times higher odds of being vulnerable in language and cognitive development, and 7.6 times higher odds of being vulnerable in communication skills and general knowledge, compared to children without teacher-reported UDNs (see Table 4).

**Table 4:** Results of unadjusted and adjusted logistic regressions of the association between UDNs and developmental vulnerability

Domains	Unadjusted OR (95% CI)	Adjusted OR (95% CI)
Overall vulnerability	9.802 (8.881–10.819)	8.434 (7.601–9.358)
Physical health & well-being	9.957 (9.184–10.794)	8.272 (7.588–9.019)
Social competence	6.930 (6.399–7.506)	5.380 (4.926–5.877)
Emotional maturity	5.812 (5.367–6.295)	4.584 (4.198–5.007)
Language and cognitive development	8.557 (7.897–9.273)	6.582 (6.030–7.185)
Communication skills & general knowledge	8.876 (8.190–9.621)	7.559 (6.909–8.270)

*Note.* All analyses were statistically significant at *p* < .001. Adjustment was performed for age, sex, special needs, E/FSL, and area-level SES; OR = odds ratio; CI = confidence interval

## Discussion

In this study of children from across Canada, teacher-reported UDNs were associated with developmental vulnerability on the EDI at the time of school entry. Children with UDNs had greater odds of being vulnerable on all of the EDI developmental domains compared to children without UDNs. Children with UDNs had 4.6 to 8.3 times higher odds of being vulnerable on the EDI domains compared to their non-UDN peers. The odds of developmental vulnerability were particularly high for physical health and well-being, language and cognitive development, and communication skills and general knowledge. These odds are comparable to those experienced by children who have a special needs designation [45, 46].

To our knowledge, this is the first study that examined teacher-reported UDNs and their association with developmental health. Our findings, indicating strong associations between UDNs and child development, suggest that teachers who identify UDNs in their students might be in a good position to advocate for oral healthcare and provide support for concurrent developmental issues. This is particularly important when considering that many children may experience a low access to routine dental care, even in a country like Canada which provides free basic health care [47, 48]. Evidence of a strong association between UDNs and children’s developmental health at school entry in all areas of development may encourage teachers to report and follow up on observed dental needs. This is particularly relevant to children with developmental disorders, such as ASD, who tend to face more obstacles in accessing care compared to typically-developing children [21]. Even though our study was not able to examine a causal pathway, our findings suggest that if a lack of adequate oral health does contribute to developmental vulnerability and is combined with a lack of access to dental care, it may have a detrimental impact on trajectories of both oral and developmental health of children throughout their lifespan.

Our study demonstrated that having UDNs was associated with the highest odds of being vulnerable in physical health and well-being, followed by communication skills and general knowledge. There are various possible explanations for these findings. Chronic pain from untreated dental caries could impact a child’s everyday functioning, including such activities as eating, sleeping, and concentrating on tasks in and outside of the classroom [49]. In addition, persistent toothache may impact speech, from difficulty opening one’s mouth, which would lead to a decreased ability to effectively communicate. UDNs could also increase emotional stress [50], which could manifest itself as either internalizing or externalizing behaviours. Additionally, being unable to concentrate in class from oral pain could decrease knowledge gain and retention, possibly impacting language and cognitive development [28]. Furthermore, severe untreated dental problems have been associated with hearing loss [51, 52]. Stereocilia (hair cells necessary for hearing) can incur damage should bacteria from dental problems enter the bloodstream, causing inflammation and narrowing of the inner ear’s blood vessels [53]. Hearing loss could then result in both expressive and receptive communication problems, as the child may face difficulty in articulating words and understanding speech. Hearing loss could also impact several other developmental domains, including social competence, language and cognitive development, and communication skills and general knowledge. This may also affect their emotional development (e.g., self-esteem), which could again result in lower social competence and communication skills (e.g., underdeveloped conversational skills, antisocial behaviour) [54, 55].

Oral health is an important part of a child’s overall health and well-being. Findings from our study suggest teacher-reported UDNs may be a marker for various developmental needs which schools and health systems might address.

### Strengths and limitations

Our study had a number of strengths including the population-based design and large sample size. Combined with fitting the regression models, this allowed us to account for multiple individual-level demographic and area-level sociodemographic factors. The study’s results indicate that UDNs are likely to be a proxy for potential deeper, more comprehensive developmental challenges, and suggest the need for further research in this area. As children with UDNs tend to live in neighbourhoods with lower SES, it is impossible to assess with our database whether the UDNs are simply associated with poverty or a result of it. Prospective studies are needed in order to enable us to disentangle the causal relationships between UDNS, special needs, SES, and developmental challenges.

One limitation is that the EDI questionnaire did not require the teacher to specify the type of UDN. UDNs may include dental caries, periodontal disease, or yellow teeth, and can have a wide range of severity. Collecting this information in future questionnaires may provide more specificity on how UDNs are associated with developmental health. For example, a study by Jackson and colleagues [56] found that absences from school resulting from dental pain were associated with poorer school performance, whereas absences for routine dental care were not. Also, the number of children with UDNs was relatively low compared to the burden of oral health issues identified in the literature. One reason for this may have been in the way the question was asked. Teachers were asked to identify any issues the child may have that *interfere* with their ability to function in the classroom. It is possible that other children in the classroom also had dental needs, but they may not have been severe enough to interfere with their ability to take part in activities in the classroom or may already have been addressed and are therefore no longer an issue. While a more general term of “dental needs” may be a more reliable indicator of oral health, it might be a less feasible teacher-reported item than “unaddressed” needs. We believe that having UDNs be reported by teachers in this context is more informative as it captures the children for whom dental issues are potentially having a negative impact on their learning. Many children have dental caries, but they are not problematic for most of them.

Another limitation is that we may not have accounted for all potential confounders of UDNs. Although we were able to control for several important variables in our analyses, information on other potential confounders was unavailable, such as information on children’s dietary intake, household income, parental employment status, prolonged bottle use, as well as various maternal and paternal characteristics. While the ability to account for these would improve the understanding of the confounders in our study, from the public health perspective, our findings represent a realistic account of information available to health service to act upon, and therefore are still valuable. Regardless of confounders, UDNs recognized by teachers are associated with children’s developmental health at school entry and therefore are an important indicator of developmental needs. Finally, there were large differences between provinces in the number of children from whom EDI information had been collected and two provinces (New Brunswick and Prince Edward Island) had to be excluded from the analyses because no EDIs were collected during the period under study. Therefore, there is a potential risk of underrepresentation of children with UDNs in certain parts of the country and results may not be generalizable to all Canadian children.

## Conclusion

In this study an association between teacher-reported UDNs and children’s developmental health at school entry was identified across all developmental domains, suggesting that UDNs may be an important marker for developmental vulnerability. Teacher-reported UDNs could prompt the school system and health professionals to consider what developmental needs a child might be experiencing across developmental domains. Considering that vulnerability in kindergarten is highly predictive of later academic problems, teacher identification of UDNs, combined with oral and developmental interventions, might positively impact children’s future health, developmental, and educational outcomes.

## Supplementary information

**Additional file 1: Table S1.** Committees and governments that approved the study protocol

## Data Availability

The dataset analysed during the current study is not publicly available due to multiple jurisdictional privacy restrictions, but it is available at the host institution.

## Abbreviations

BLR: Binary logistic regression
CCHICS: Canadian Children’s Health in Context Study
CI: Confidence interval
E/FSL: English/French as a Second Language
EDI: Early Development Instrument
OR: Odds ratio
SES: Socioeconomic status
SHNs: Special health needs
SPSS: Statistical Package for Social Sciences
UDNs: Unaddressed dental needs

## References

[1] Kassebaum NJ, Bernabé E, Dahiya M, Bhandari B, Murray CJL, Marcenes W (2015). Global burden of untreated caries: a systematic review and metaregression. J Dent Res.

[2] Petersen Poul Erik (2003). The World Oral Health Report 2003: continuous improvement of oral health in the 21st century - the approach of the WHO Global Oral Health Programme. Community Dentistry and Oral Epidemiology.

[3] Canadian Institute for Health Information (2013). Treatment of Preventable Dental Cavities in Preschoolers: A Focus on Day Surgery Under General Anesthesia.

[4] BC Ministry of Health, Population and Public Health Division. British Columbia Dental Survey of Kindergarten Children: A Provincial and Regional Analysis, 2015/2016. Gov Br Columbia; 2017.

[5] OECD. Health at a glance 2011: OECD Publishing; 2011. 10.1787/health_glance-2011-en.

[6] Mariño RJ, Calache H, Whelan M (2014). Socio-demographic profile of child and adolescent users of oral health services in Victoria. Aust Cad Saude Publica.

[7] McMahon AD, Elliott L, Macpherson LM (2018). Inequalities in the dental health needs and access to dental services among looked after children in Scotland: a population data linkage study. Arch Dis Child.

[8] Östberg A-L, Kjellström AN, Petzold M (2017). The influence of social deprivation on dental caries in Swedish children and adolescents, as measured by an index for primary health care: the care need index. Community Dent Oral Epidemiol.

[9] Poon BT, Holley PC, Louie AM, Springinotic CM (2015). Dental caries disparities in early childhood: A study of kindergarten children in British Columbia. Can J Public Health Rev Can Sante Publique.

[10] da Fonseca MA, Avenetti D (2017). Social determinants of pediatric oral health. Dent Clin N Am.

[11] National Center for Health Statistics. Health, United States, 2010. Hyattsville, MD NCHS; 2011:563. 21634072

[12] Grignon M, Hurley J, Wang L, Allin S (2010). Inequity in a market-based health system: evidence from Canada’s dental sector. Health Policy Amst Neth.

[13] Darmawikarta D, Chen Y, Carsley S (2014). Factors associated with dental care utilization in early childhood. Pediatr.

[14] Hakeberg M, Wide BU. Self-reported oral and general health in relation to socioeconomic position. BMC Public Health. 2017;18. 10.1186/s12889-017-4609-9. 10.1186/s12889-017-4609-9 PMC5530538 28747180

[15] Sabbah W., Tsakos G., Chandola T., Sheiham A., Watt R.G. (2007). Social Gradients in Oral and General Health. Journal of Dental Research.

[16] Dworet D, Bennett S (2002). A view from the north: special education in Canada. Teach Except Child.

[17] Adyanthaya A, Sreelakshmi N, Ismail S, Raheema M (2017). Barriers to dental care for children with special needs: general dentists’ perception in Kerala, India. J Indian Soc Pedod Prev Dent.

[18] Mandić J, Jovanović S, Mandinić Z (2018). Oral health in children with special needs. Vojnosanit Pregl.

[19] Nelson LP, Getzin A, Graham D, et al. Unmet Dental Needs and Barriers to Care for Children with Significant Special Health Care Needs. https://www.ingentaconnect.com/content/aapd/pd/2011/00000033/00000001/art00006. Published February 2011. Accessed 30 Nov 2018. 21406145

[20] Dao LP, Zwetchkenbaum S, Inglehart MR (2005). General dentists and special needs patients: does dental education matter?. J Dent Educ.

[21] Lai Bien, Milano Michael, Roberts Michael W., Hooper Stephen R. (2011). Unmet Dental Needs and Barriers to Dental Care Among Children with Autism Spectrum Disorders. Journal of Autism and Developmental Disorders.

[22] Anders PL, Davis EL (2010). Oral health of patients with intellectual disabilities: a systematic review. Spec Care Dentist.

[23] Shepherd MA, Nadanovsky P, Sheiham A (1999). The prevalence and impact of dental pain in 8-year-old school children in harrow. England Br Dent J.

[24] Gilchrist F, Marshman Z, Deery C, Rodd HD (2015). The impact of dental caries on children and young people: what they have to say?. Int J Paediatr Dent.

[25] Sheiham A (2005). Oral health, general health and quality of life. Bull World Health Organ.

[26] Acs G, Lodolini G, Kaminsky S, Cisneros GJ (1992). Effect of nursing caries on body weight in a pediatric population. Pediatr Dent.

[27] Blumenshine SL, Vann WF, Gizlice Z, Lee JY (2008). Children’s school performance: impact of general and oral health. J Public Health Dent.

[28] Guarnizo-Herreño CC, Wehby GL (2012). Children’s dental health, school performance and psychosocial well-being. J Pediatr.

[29] Seirawan H, Faust S, Mulligan R (2012). The impact of oral health on the academic performance of disadvantaged children. Am J Public Health.

[30] Keating DP, Hertzman C (1999). Developmental health and the wealth of nations.

[31] Marmot M, Friel S, Bell R, Houweling TA, Taylor S (2008). Closing the gap in a generation: health equity through action on the social determinants of health. Lancet.

[32] Davies Scott, Janus Magdalena, Duku Eric, Gaskin Ashley (2016). Using the Early Development Instrument to examine cognitive and non-cognitive school readiness and elementary student achievement. Early Childhood Research Quarterly.

[33] Janus Magdalena, Offord David R. (2007). Development and psychometric properties of the Early Development Instrument (EDI): A measure of children's school readiness. Canadian Journal of Behavioural Science / Revue canadienne des sciences du comportement.

[34] Muhajarine N, Puchala C, Janus M (2011). Does the EDI equivalently measure facets of school readiness for aboriginal and non-aboriginal children?. Soc Indic Res.

[35] Janus Magdalena, Zeraatkar Dena, Duku Eric, Bennett Teresa (2018). Validation of the Early Development Instrument for children with special health needs. Journal of Paediatrics and Child Health.

[36] Duku E, Janus M, Brinkman S (2015). Investigation of the cross-national equivalence of a measurement of early child development. Child Indic Res.

[37] Government of Canada SC. Population estimates on July 1st, by age and sex. https://www150.statcan.gc.ca/t1/tbl1/en/tv.action?pid=1710000501. Published December 27, 2017. Accessed 12 June 2019.

[38] Government of Canada SC. Back to school... by the numbers. https://www.statcan.gc.ca/eng/dai/smr08/2018/smr08_220_2018. Published August 27, 2018. Accessed 12 June 2019.

[39] Janus Magdalena, Brownell Marni, Reid-Westoby Caroline, Bennett Teresa, Birken Catherine, Coplan Robert, Duku Eric, Ferro Mark A, Forer Barry, Georgiades Stelios, Gorter Jan Willem, Guhn Martin, Maguire Jonathon L, Manson Heather, Pei Jacqueline, Santos Rob (2018). Establishing a protocol for building a pan-Canadian population-based monitoring system for early childhood development for children with health disorders: Canadian Children’s Health in Context Study (CCHICS). BMJ Open.

[40] Janus M, Duku E (2007). The school entry gap: socioeconomic, family, and health factors associated with children’s school readiness to learn. Early Educ Dev.

[41] Janus M, Reid-Westoby C. Monitoring the development of all children: the Early Development Instrument. In: Moreno T, editor. Early childhood matters. The Hague, Netherlands: Bernard van Leer Foundation; 2016. p. 40–5.

[42] Guhn M, Janus M, Enns J (2016). Examining the social determinants of children’s developmental health: protocol for building a pan-Canadian population-based monitoring system for early childhood development. BMJ Open.

[43] Forer B, Minh A, Enns J, et al. A Canadian neighborhood index for socioeconomic status associated with early child development. Child Indic Res. 2019. 10.1007/s12187-019-09666-y.

[44] Corp IBM (2017). IBM SPSS statistics for windows, version 25.0.

[45] Janus M (2011). Transition to School: Child, family, and community-level determinants. Transitions to Early Care and Education. Educating the Young Child. Vol 3.

[46] Janus M, Duku E, Hughes D (2010). Canadian Council for Learning. Patterns of school readiness among selected groups of Canadian children: children with special needs and children with diverse language backgrounds.

[47] Canadian Dental Association (2010). Position paper on access to Oral Care for Canadians.

[48] Canadian Academy of Health Sciences. Improving Access to Oral Health Care for Vulnerable People Living in Canada.; 2014.

[49] Canadian Dental Association. Early Childhood Caries. https://www.cda-adc.ca/en/about/position_statements/ecc/. Published 2010. Accessed 7 March 2019.

[50] Lumley MA, Cohen JL, Borszcz GS (2011). Pain and emotion: a biopsychosocial review of recent research. J Clin Psychol.

[51] Lawrence HP, Garcia RI, Essick GK (2001). A longitudinal study of the association between tooth loss and age-related hearing loss. Spec Care Dentist..

[52] Wu C-S, Yang T-H, Lin H-C, Sheu J-J, Chu D (2013). Sudden sensorineural hearing loss associated with chronic periodontitis. Otol Neurotol.

[53] Plotnick B. Oral health and hearing loss. Healthy Hearing. https://www.healthyhearing.com/report/50978-Oral-health-and-hearing-loss. Published 2013. Accessed 8 March 2019.

[54] Champion J, Holt R (2000). Dental care for children and young people who have a hearing impairment. Br Dent J.

[55] Maharani DA, Adiatman M, Rahardjo A, Burnside G, Pine C (2017). An assessment of the impacts of child oral health in Indonesia and associations with self-esteem, school performance and perceived employability. BMC Oral Health.

[56] Jackson SL, Vann WF, Kotch JB, Pahel BT, Lee JY (2011). Impact of poor oral health on children’s school attendance and performance. Am J Public Health.

