# Supplementary material for: Association between severe unaddressed dental needs and developmental health at school entry in Canada: a cross-sectional study
Source: BMC Pediatr. 2019 Dec 7;19:481. doi: 10.1186/s12887-019-1868-x (PMC6898915; doi:10.1186/s12887-019-1868-x)
Supplement: Supplementary file 1 — Additional file 1: Table S1. Committees and governments that approved the study protocol [file 12887_2019_1868_MOESM1_ESM.docx]

Supplementary Table 1. Committees and governments that approved the study protocol

| Ethics committees: |
| --- |
| Hamilton Integrated Research Ethics Board (HiREB) |
| University of Manitoba Health Research Ethics Board |
| Provincial/territorial governments who granted permission to collect EDI data: |
| Alberta   - Alberta Education - Human Resources and Social Development Canada |
| British Columbia   - Ministry of Children and Family Development |
| Manitoba   - Healthy Child Manitoba Office |
| New Brunswick   - Human Resources and Social Development Canada - Department of Social Development, Province of New Brunswick |
| Newfoundland and Labrador   - Government of Newfoundland and Labrador, Department of Education, Division of Early Childhood Learning |
| Northwest Territories   - Government of the Northwest Territories, Department of Education, Culture and Employment |
| Nova Scotia   - The Nova Scotia Department of Education - Human Resources and Social Development Canada |
| Ontario |
| - Ministry of Children and Youth Services (2004-2012) |
| - Ministry of Education (2015) |
| Prince Edward Island   - Government of Prince Edward Island, Minister of Education, Early Learning and Culture |
| Québec   - Institut de la statistique du Québec - Human Resources and Social Development Canada |
| Saskatchewan   - Province of Saskatchewan, Minister of Education - Human Resources and Social Development Canada |
| Yukon   - Government of Yukon – Department of Education |
